# A disaster victim identification workshop focused on forensic odontology using embalmed human remains

**DOI:** 10.1007/s00414-022-02790-5

**Published:** 2022-03-02

**Authors:** Johann Zwirner, Warwick Duncan

**Affiliations:** 1 Department of Anatomy, University of Otago, Dunedin, New Zealand; 2 Institute of Legal Medicine, University Medical Center Hamburg-Eppendorf, Hamburg, Germany; 3 Institute of Legal Medicine, University of Leipzig, Leipzig, Germany; 4 Sir John Walsh Research Institute, University of Otago, Dunedin, New Zealand

**Keywords:** Cadaver, DVI, Embalming, Forensic odontology, Workshop

## Abstract

A high number of victims of mass casualty incidences are identified through their teeth. While forensic odontologists need to have a complex skillset during a disaster victim identification (DVI) response, hands-on training opportunities are rare. In countries with very limited forensic casework, such as New Zealand, many forensic odontologists find it difficult to achieve the number of annual forensic dental identifications required to maintain their credentialling. This report details the development of a hands-on forensic odontology-focused DVI workshop using human Crosado-embalmed remains. Anonymous participant evaluations, including five-point Likert and open-ended items, were performed in both years the workshop was held. A total of 10 and 17 participants, predominantly dentists, attended the workshop in 2020 and 2021, respectively. Participant feedback was extremely positive. Likert items were statistically similar between participants in both years. Open-ended items revealed positive feedback regarding the use of cadaveric remains, the gained hands-on experience, or the teamwork aspect. Participants who attended the workshop in both years commented on the positive aspect of repetition to cement their skills. As areas of improvement, participants named (for example) time management and the number of portable X-ray devices, leading to changes that were implemented in 2021. Moreover, the participants expressed interest to further their skills on decomposed, burnt, and fragmented human remains, which for ethical reasons has yet to be implemented. The DVI workshop described here, using embalmed human remains, provides an opportunity to add dental identifications toward annual credentialling requirements for forensic odontologists. Participants rated the course to be excellent overall and highly relevant for their role. For future workshops, there is an interest to include further aspects of the DVI response such as fingerprinting or police work as well as remains, which are altered due to natural or physical reasons.

**Supplementary Information:**

The online version contains supplementary material available at 10.1007/s00414-022-02790-5.

## Introduction

Forensic odontology is one of the primary identifiers in man-made or natural mass casualty incidences [1]. The sole investigation of dental remains by experienced forensic odontologists led to the identification of at least 60% of victims of the Bali bombings in 2002 [2] or as many as 79% of the Boxing Day Tsunami victims in Thailand in 2004 [3].

In New Zealand, all forensic odontologists must be practicing general dental practitioners or dental specialists and must also be a credentialled member of the New Zealand Society of Forensic Odontology (NZSFO; see: https://nzsfo.org.nz/). The society oversees the annual certification of members in the General Forensic Odontology Scope of Practice, which allows them to conduct routine human identification casework as directed by the coronial services of New Zealand. Experienced forensic odontologists may subsequently apply to the NZSFO for credentialling in the extended scope of practice of DVI. Only those who have completed training and are credentialled in this extended scope are able to participate in DVI operations in New Zealand.

A questionnaire sent to 50 internationally known forensic dentists identified that 31% of the participants were lacking casework and 17% answered that only limited hands-on courses were available to gain experience in the field [4]. In comparatively peaceful countries such as New Zealand, with limited numbers of forensic dental identifications and unequal distribution of a small population across a wide area of land, it can be exceptionally challenging for forensic odontologists to accumulate the annually needed number of identifications to maintain their license. Recent events in Christchurch, such as the earthquake in 2011 with 181 deaths [5] or the mosque shooting in 2019 with 51 deaths [6], demonstrated the need for well-trained personnel to identify the victims. Following the loss of an Air New Zealand aircraft on Mt. Erebus in Antarctica in 1979, the NZSFO was formed in 1983 [7], with the responsibility to the New Zealand Chief Coroner for both the regular identification of human remains throughout the country, as well as the coordination of forensic odontology teams contributing to a DVI response at the national and international level [5]. For this, an elaborate DVI odontology readiness plan is in place, which follows the guidelines of the International Organization for Forensic Odonto-Stomatology, INTERPOL, and the Australian Society of Forensic Odontology [5]. Given the lack of hands-on DVI courses using cadaveric remains in the Pacific region, we have developed a workshop focused on the identification of dental remains. This manuscript summarizes the workshop objectives and design as well as the student evaluations of the first 2 years after its establishment. Semi-flexible embalmed human tissues were used [8,9]; these have previously proven suitable for both undergraduate and postgraduate medical and dental teaching. The cadaver-based DVI workshop design presented here provides an excellent opportunity to gain casework experience in a safe environment and accumulate identifications that count toward annual credentialling, which is of interest for any forensic dental society worldwide.

## Material/methods

### Ethical approval

A minimal risk ethical approval for this teaching workshop was granted by the Departmental Ethics Committee of the Department of Anatomy and confirmed by the Human Ethics Committee of the University of Otago (reference number: D21/373).

### Workshop objectives

The main objective of the workshop in 2020 was to enable dental and auxiliary dental staff as members of the NZSFO to perform a maximum of eight forensic dental identifications, which counted towards the annual credentialling as part of their continuing professional development (CPD). In addition to this objective, the workshop in 2021 intended to foster team building between different professionals involved in a DVI scenario. Moreover, course registrations were permitted for final year undergraduate dental students with an interest in forensic odontology, allowing them to explore this career path.

### Workshop design

**Fig. 1:**
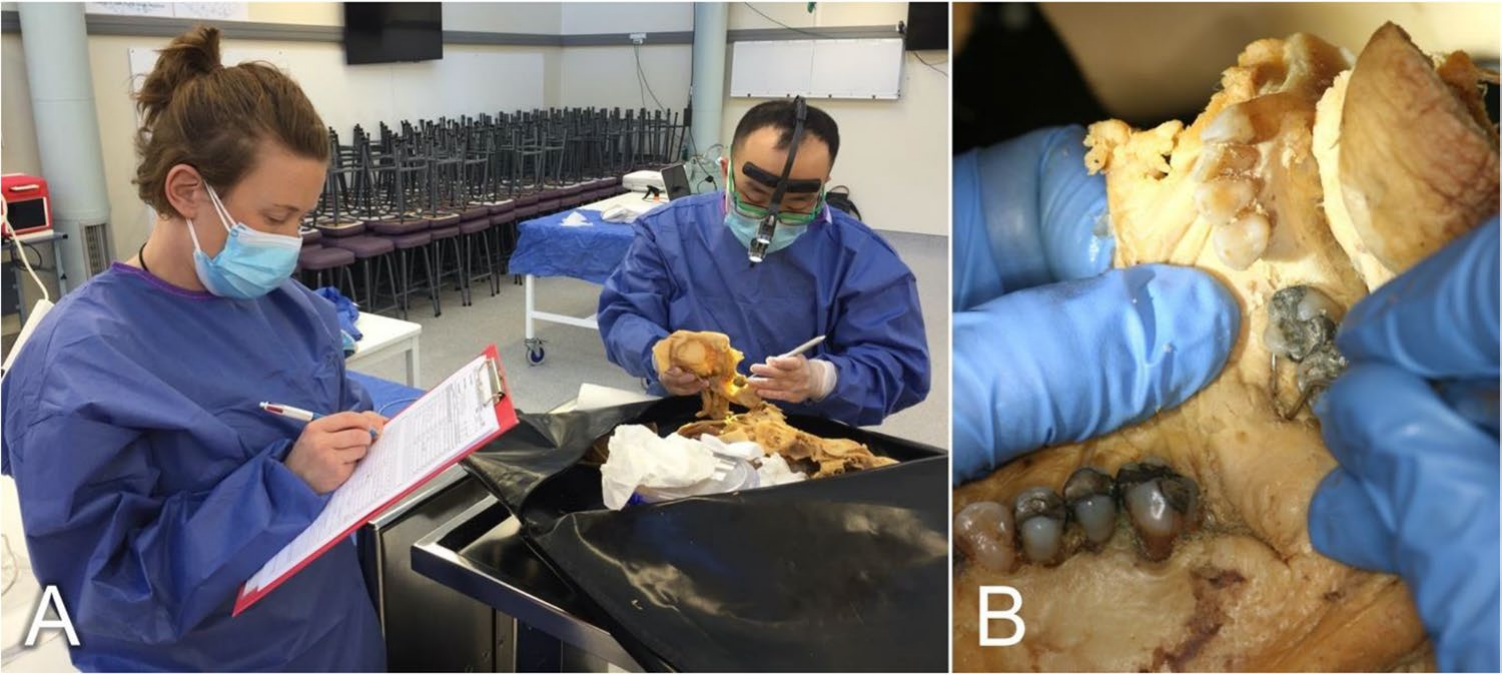
**A** Postmortem charting is done in pairs using basic dental examination instruments and INTERPOL forms. **B** Crosado-embalmed human remains are shown during the dental charting

The core of the workshop is the performance of simulated forensic dental identifications on semi-flexible Crosado-embalmed [8] human cadavers, which had been donated to the Department of Anatomy Dunedin of the University of Otago for research and teaching purposes. The fixative components and respective amounts, which were used for the cadavers of this workshop, are depicted in Table 1. Prior to the workshop, the cadavers had been used for medical and dental undergraduate teaching. In 2020, the oral cavities were kept intact, while in 2021, all heads were cut in the sagittal plane, which divided the oral cavities into two symmetrical pieces. Cadavers made available through the Body Bequest program at the University of Otago are anonymous and do not include antemortem dental records. Accordingly, the workshop organizers created simulated dental records, including radiographs and dental charting of simulated dental treatment, which participants were required to interpret before commencing postmortem examinations. The participants were then asked to perform postmortem dental charting and take intraoral dental radiographs for all the embalmed cadaveric remains and to reconcile these against the simulated antemortem records with the object being to produce a report to the coroner. To comply with work practices used in a real DVI scenario, the participants had to perform the charting on INTERPOL forms using the FDI notation system (Fig. 1). The NZSFO charting rules for DVI operations, which are in accordance with INTERPOL recommendations, are depicted in Supplementary Fig. 1. Introductory seminars on DVI and charting conventions, as well as written guides and a template for the production of the coronial report, were provided at the start of the workshop. The workshop was set up as a “closed” DVI scenario, with eight simulated antemortem records as described above being available to the participants in 2020 (including only the cadavers used by the participants during the workshop) and 16 simulated antemortem records in 2021; the latter included all cadavers used in the 2021 workshop plus eight additional records from other cadavers, since the exercise scenario stated that not all of the victims bodies had been recovered. Examples of a simulated newspaper article used to establish the scenario, and the simulated antemortem dental records, are shown in Supplementary Figs. 2 and 3.

**Table 1:** Fixative components of the Crosado fluid are reported for a 70 kg cadaver as described in Crosado et al. [8]. Arquad 2HT, dimethyl di(hydrogenated tallow) ammonium chloride

	**Fixative component**	**Amount**
Fixation solution	Ethanol	40 L
	Glycerin	10 L
	Water	10 L
	Phenoxyethanol (90%)	5 L
	Formaldehyde (37%)	1.25 L
Additional brain fixation solution	1:5 ratio of formaldehyde (37%) and fixation fluid	20 mL
Storage solution	Water	10 L
	Phenoxyethanol (90%)	150 mL
	Arquad 2HT	40 mL

**Fig. 2:**
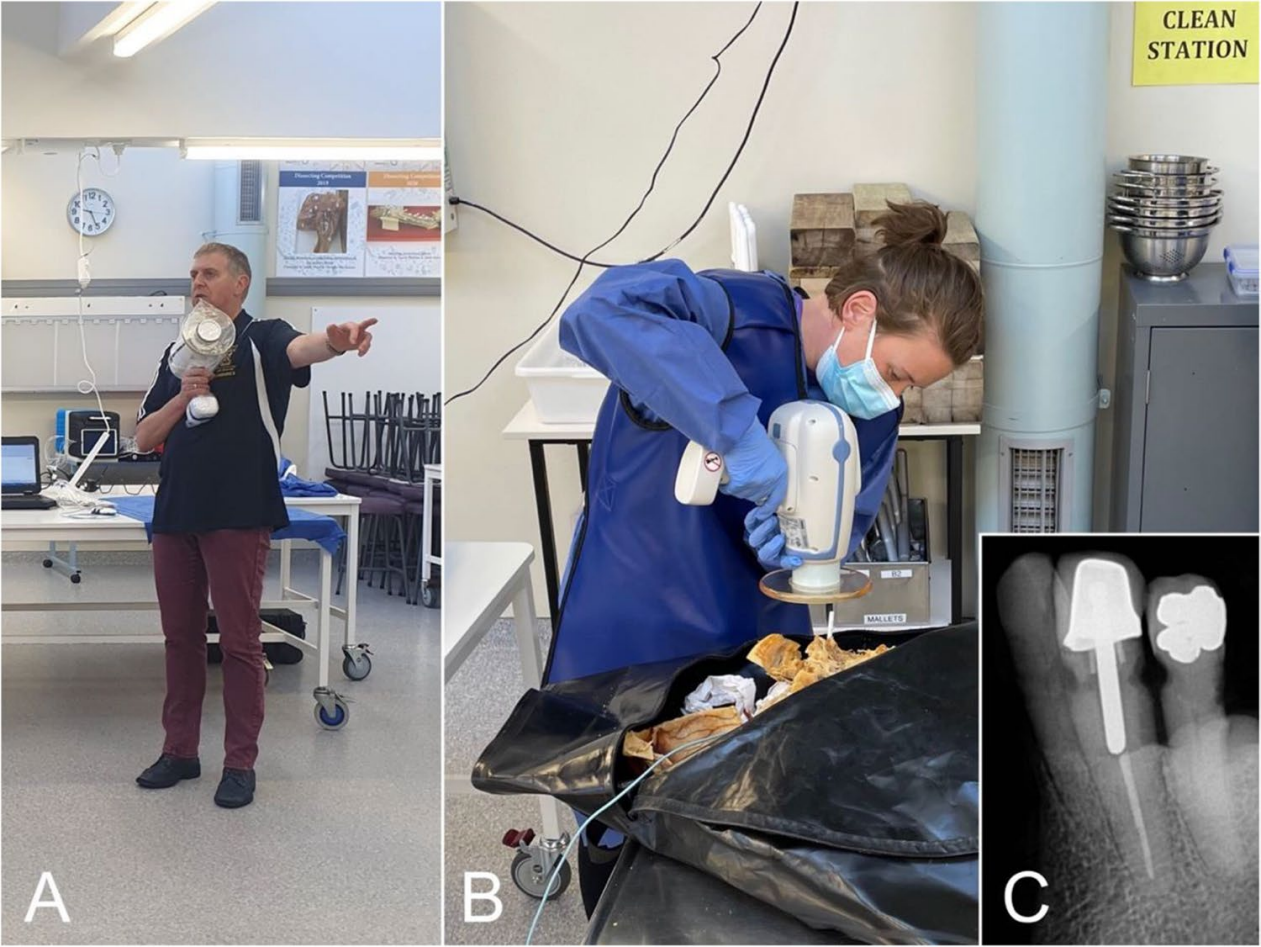
**A** The instructor introduces the participants to how to use the hand-held X-ray units (NOMADs) in a safe way. **B** A participant uses the NOMAD on embalmed human remains. **C** An X-ray image produced by the participants using the NOMAD device is depicted

For the postmortem radiography, portable battery-powered hand-held X-ray units (NOMADs, Aribex, East Orem, USA) with laptops and basic dental examination instruments were utilized (Fig. 2). The participants were asked to bring their own laptops for report writing, clinical cameras, headlamps, and loupes.

**Fig. 3:**
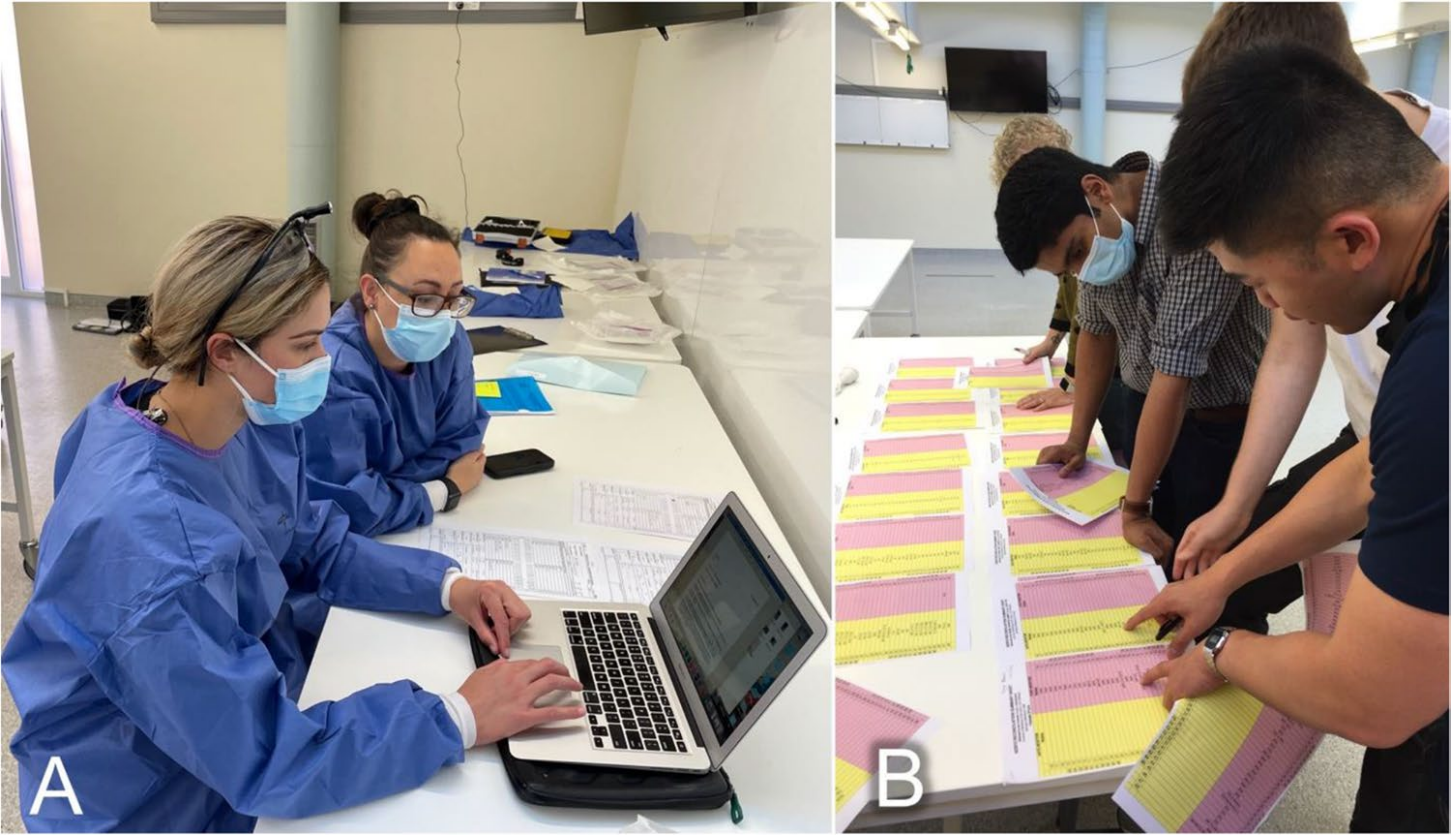
**A** Clean write-up area was established during the course as commonly done in real DVI responses. **B** The participants reconcile ante- and postmortem chartings using official summary sheets of the NZSFO

Anonymous participant evaluations were performed after both courses. For data entry, a clean write-up area was established in the dissection room of the Department of Anatomy Dunedin, where the workshop took place (Fig. 3). Only completed coronial reports were counted toward annual credentialling. Participant suggestions of the 2020 workshop were implemented in the course design for the following year. The time spent with certain tasks of the workshops in 2020 and 2021 is depicted in Table 2. Differences between the two workshops are presented in Table 3.

**Table 2:** The time committed to different tasks of the workshop is depicted for 2020 and 2021

	2020		2021	
	Hours/day time	Task/event	Hours/day time	Task/event
Day 1	0.5	Workshop introduction and lab orientation	0.5	Workshop introduction and lab orientation
	6.75	Postmortem charting	1.5	DVI lecture
	Evening	Socializing event	4	Antemortem charting
Day 2	8	Complete postmortem charting	6.75	Postmortem charting
		Antemortem charting		
		Reconciliation and report writing		
			Evening	Socializing event
Day 3		8	Complete postmortem charting. Reconciliation and report writing	
Total CPD hours	15.25		20.75	

*CPD*, continuing professional development.

**Table 3:** Differences of the two workshops in 2020 and 2021 are depicted

	2020	2021
Participants	10	17
Cadavers (= postmortem examinations)	8	8
Antemortem examinations	8	16
NOMAD X-ray units	2	3
Oral cavities	Intact	Cut in sagittal plane
Participants	Dentists and dental auxiliary staff	Dentists, dental auxiliary staff, dental students, and police

### Workshop evaluation

After the course, the participants were invited to participate in an anonymous workshop evaluation including 11 items (I–XI). The participants were asked to answer the following five items on a five-point Likert scale: (I) To what extent did this workshop increase your understanding of the topic?; (II) To what extent did this workshop provide content relevant to your role in forensic odontology?; (III) To what extent did this workshop provide strategies/tools that you will use in your role?; (IV) To what extent did this workshop provide content that was organized and easy to follow?; (V) To what extent did this workshop provide course materials that were relevant and useful? The participants were able to choose between the following item scores for the items I–V: “To a great extent” (1), “To some extent” (2), “To a slight extent” (3), “To a very slight extent” (4), and “Not at all” (5). Item VI consisted of the following question on a five-point Likert scale: How would you rate this workshop overall? The following item scores could be chosen for item VI: “Excellent” (1), “Good” (2), “Average” (3), “Fair” (4), and “Poor” (5). Items VII–XI were open ended: (VII) Name one positive outcome that you gained from the workshop.; (VIII) What were the best things about this workshop for you?; (IX) What was the one thing that you will take away and use after this workshop?; (X) How could we improve this workshop?; (XI) What topics or skills would you like addressed in future workshops?

### Statistical analysis

Excel version 16.54 (Microsoft Corporation, Redmond, WA, USA) and GraphPad Prism version 9 (GraphPad Software, La Jolla, CA, USA) were used for statistical analyses. Two-tailed Mann–Whitney *U* tests were performed to compare the Likert scale items. Two sets of comparisons were made: Firstly, matching items were compared between all participants of the workshops in 2020 and 2021. Secondly, only answers of second-year attendees in 2021 were compared to the answers of all participants of the year before. *P* values equal to or smaller than 0.05 were considered statistically significant.

## Results

### Answers to five-point Likert scale items 2021 and 2022

Participation in the voluntary workshop evaluation was high, with 90 and 100% in 2020 and 2021, respectively. Table 4 depicts the mean scores for items I to VI separately for all participants in 2020 and 2021, as well as participants who attended both years (second-time participants in 2021). Mean scores for the item I indicated that the workshop increased the participant’s understanding of the topic to a great extent. According to the participant’s rating of item II, the workshop greatly provided content relevant to the role of the participant in forensic odontology. For item III, mean scores indicated that the workshop, to a great extent, provided strategies and tools that the participants could use in their role. Mean scores for item IV showed that the content of the workshop was considered to be highly organized and easy to follow by the participants. Following the rating for item V, the course materials are highly relevant and useful to the participants. Means scores for item VI showed that the overall rating of the course by the participants was excellent.

**Table 4:** The mean scores for items I to VI are depicted for the different groups

Items	I	II	III	IV	V	VI
2020 [all]	1.222	1.000	1.000	1.111	1.111	1.111
2021 [all]	1.588	1.294	1.353	1.353	1.118	1.176
2021 [2nd time participants]	1.714	1.143	1.286	1.143	1.000	1.143

**Fig. 4:**
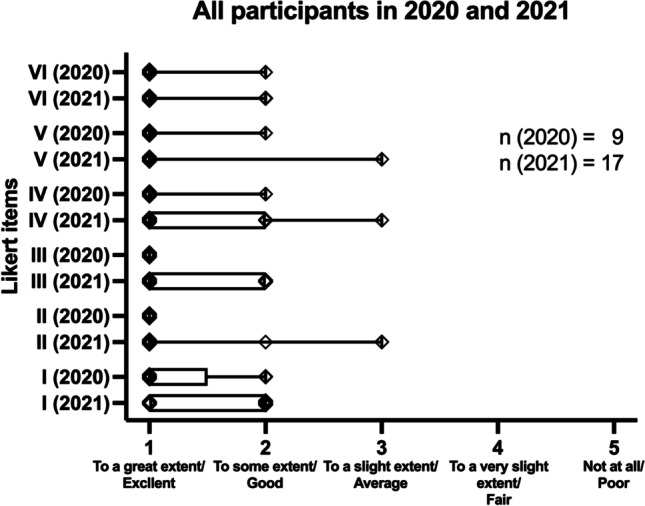
Boxplots with whiskers showing the minima and maxima including all points (small squares) for the seven different 5-point Likert items of the study evaluation of all participants in 2020 and 2021 are depicted

The results of the Mann–Whitney *U* tests indicated no statistically significant differences between the evaluation results of all participants in 2020 and 2021. Also, no significant differences were detected when comparing the results of all participants in 2020 with the ones in 2021 who attended both years. A summary of the test results is given in Table 5. A graphical summary of the results for the comparisons between all participants in 2020 and 2021, as well as between all participants in 2020 and participants in 2021 who attended both courses, is given in Fig. 4.

**Table 5:** A summary of the Mann–Whitney *U* test results is given

	All participants in 2020 and 2021					
Items	I vs. I	II vs. II	III vs. III	IV vs. IV	V vs. V	VI vs. VI
Mann–Whitney *U*	48.5	63	49.5	62	73	71.5
*P* value	0.110	0.372	0.063	0.339	> 0.999	> 0.999
	**All participants in 2020 and 2nd time participants in 2021**					
Items	**I vs. I**	**II vs. II**	**III vs. III**	**IV vs. IV**	**V vs. V**	**VI vs. VI**
Mann–Whitney *U*	16	27	22.5	30.5	28	30.5
*P* value	0.126	0.438	0.175	> 0.999	> 0.999	> 0.999

### Answers to open-ended items VII to XI in 2020 and resulting adaptations to the workshop structure in 2021

With regards to the volume of different answers for items VII to XI, only examples will be presented here. A summary of all given answers for items I to XI is given in Supplementary Table 1 for both years of the workshop evaluation. In six of the nine answers regarding one positive outcome of the workshop in 2020 (item VII), the participants used the word “experience.” As the best things of the workshop (item VIII) participants, named the “hands-on” experience, the “mentor style of support,” or the opportunity to work on “human cadaver remains.” The one thing to take away from this workshop (item IX) was the “charting experience” and the importance of a “systematic” approach to the identification to save “time down the track.” The participant responses to items X and XII in 2020 and how they were implemented in the workshop design by the course faculty in 2021 are depicted in Table 6.

**Table 6:** The participant answers to items X (“How could we improve this workshop?”) and XI (“What topics or skills would you like addressed in future workshops?”) in 2020 and the implementation in the workshop design in the following year are shown

Participant responses to item X in 2020	Implementation in workshop 2021
“Twists in cases”	Increasing the number of antemortem records to make identifications more challenging
“Have another day so paper work […can] be […done] on course. My partner has not been available since to do it.”	Adding another day to the workshop to give more time for individual tasks
“Perhaps 8 cases were too many for a 2-day workshop”	
“Having more NOMAD devices for taking radiography”	Increasing number of NOMAD X-ray units from 2 to 3
Participant responses to item X in 2020	
“Fragmented and decomposed remains”	Cutting heads in half in the sagittal plane, which divided the oral cavities in two symmetrical pieces to allow for better access to the teeth
“Time for AM and reconciliation to be done as part of course”	Adding another day to the workshop to give more time for individual tasks

### Answers to open-ended items in 2021

Participants who attended the workshop for the first time appreciated the “Cases towards credentialling,” the “work around the cadavers,” to “learn […] from colleagues” as well as the “knowledge in DVI process and equipment” while participants who attended for the second time valued the “repetition” to “cement […] skills” (for item VII). For item VIII, first-time attendees named the “exposure to the cadavers” and “meeting other forensic personnel,” while second-time attendees also positively commented on the increased number of team members per cadaver. For item IX, the participants commented that it was “Great to work as a team in a safe environment to the forensic world” or the “information gained, which […they] will be able to add to over further courses or DVI.”

New participants identified the following selected points as room for improvement (item X): “teams with instructors,” “run more workshops,” more publications on the topic so “knowledge can be improved,” and “more clear outline/plan to learn and prepare before the course.” Second-time workshop attendees again highlighted the need for “more NOMAD units,” the desire to have “burnt remains” as part of the workshop, and suggested a “multi-disciplinary practice/exercise with police.” For item XI, the participants highlighted skills on “writing of reports” and the need to further increase the time for report writing as important points. Further topics or skills desired in future workshops were “instruction sheet” for the NOMAD X-ray units, the implementation of “fingerprinting, properties [and…] full DVI reports”, “More Antemortem investigations” or a “presentation from a coroner.”

## Discussion

This report detailed the objectives and design of a newly developed hands-on DVI workshop using embalmed human remains. Overall, the anonymous participant evaluation showed a great appreciation for the new workshop and pointed out room for improvement.

### A well organized and highly relevant workshop, which was rated “excellent” overall

Altogether, the two held workshops in 2020 and 2021 were rated extremely positive by the participants. The vast majority of participants gave the highest scores regarding whether the workshop increased their understanding of the topic, provided content relevant to their role, provided useful strategies and relevant course materials, or was well organized. Overall, the workshop was rated “excellent” by the participants. A statistical comparison between the answers to the Likert items in 2020 and 2021 revealed equally positive feedback with no significant differences between the two years. Comments related to the cadaveric remains were positive throughout, which shows that the participants highly value the exposure to this invaluable resource. The DVI workshop presented here stands out due to the fact that wet cadaveric tissues were used for the mock identification cases, as opposed to only hard tissues [10] without attached soft tissues such as lips, tongue, or gingiva. Crosado-embalmed cadaveric tissues are fixated with a solution containing predominantly ethanol and stored in a solution consisting of phenoxyethanol as the major component after water [8]. However, even though in the Crosado embalming, the use of formalin is reduced to a minimum without compromising the fixation result, it must not be ignored that formalin is toxic [11], allergenic [12], and potentially even carcinogenic [13,14]. Single-use latex gloves, surgical face masks, and surgical aprons were used to avoid skin contact and reduce the inhalation of formalin fumes. Moreover, the body bags were kept closed except for the head part to limit the evaporation of formalin fumes into the dissection room, and proper ventilation was assured at any time.

With regards to its biomechanical properties, Crosado-embalmed tissues are more “semi-flexible,” placing them between the rigid formaldehyde-fixed and the in vivo-like thiel-embalmed tissues [8,15]. The advantage of having a certain degree of flexibility in the tissues that are used for DVI workshops is the ability to open the oral cavity for charting purposes. Qualitatively, Crosado-embalmed tissues seem to mimic the rigor mortis, which makes this fixation method attractive for forensic teaching courses. The work with embalmed tissues in the dissection room of an anatomical department is a suitable training environment for forensic dentists, given that their daily work environment rather aims to comfort living patients than being exposed to dissected human remains. The “safe environment,” which was created in this workshop, was positively commented on in the evaluation. The workshop participants had experience levels ranging from those with years of experience, including prior participation in national-level disasters, to team members with no prior exposure to human remains. Bearing in mind the psychological challenges that are associated with forensic casework, gradual exposure to death and sensitive images as done here might help participants to build resilience [4], which is important for their own protection.

### Desire to analyze decomposed, burnt, and fragmented remains

DVI operations often involve decomposed, fragmented, and burnt remains, which significantly complicates the identification process due to commingling and cross-contamination [16]. The participant’s desire to analyze such remains is understandable and training opportunities in this regard should ideally be available to further the skill set of forensic odontologists. However, the use of decomposed, fragmented, and burnt human remains for teaching or professional development purposes has to be ethically justified. The volitional decomposition of human bodies for academic purposes is highly controversial and only done in a few countries within the “body farm” concept [17]. While some rate the study of decomposed human remains as important for forensic purposes, others find it “gruesome and grim” [17]. The specific question, which forensic odontologists should answer, is whether learning outcomes are altered by conducting forensic odontology training using teeth (hard tissues) in decomposed remains, which predominantly affects soft tissues structures in DVI responses. While the workshop faculty will consult the local ethics committee to explore the potential implementation of decomposed human remains in future DVI workshops, a thorough analysis of the educational benefit of this resource is needed. Also, it has to be clarified to what extent decomposed animal tissues are a sufficient replacement for human tissues. Information on burnt human remains is usually gathered during forensic autopsy [18] or based on anthropological studies of previously burnt bones [19]. If the remains are burnt by the researchers themselves, animal tissues are chosen over human ones due to ethical concerns [20,21]. Again, for the forensic odontology-focused DVI workshop presented here, the benefit of using burnt remains has to be ethically justified and should be further investigated in the future. Potentially, it makes a difference whether only the teeth are burnt to study their morphological change following heat exposure as has been previously published [22], or if intact body parts, including soft tissues (e.g., face, soft palate) are burnt. The fragmentation of human remains for the workshop was partly implemented in 2021 by cutting the heads in halves following the participant evaluation in 2020. However, this workshop did not include an analysis of fragmentary human remains. This seems to be achievable in future workshops from an ethical perspective, as regularly performed anatomical dissections for teaching purposes produce small tissue pieces as well. This includes bone fragments, which are, e.g., produced when the brain is retrieved from the skull. Therefore, future analysis of small fragments seems to be less challenging from an ethical perspective as opposed to decomposed or burnt remains.

### Participant evaluations are key for developing and improving the workshop in the future

Participant evaluations are considered invaluable for further developing the workshop, detecting general areas of improvement, and tailoring the workshop design to the needs of the participants. For example, the 2020 evaluation identified that the time to complete the set tasks was ambitious and that more NOMAD X-ray devices were needed. In 2021, the number of NOMAD devices was increased, and both the number of participants and workshop hours were increased without increasing the number of identifications. The evaluations highlighted the interest of the participants to include other aspects of the DVI process, such as fingerprinting or involving other professionals of the DVI response team, such as the police. Hence, the workshop could become more holistic in the future with higher numbers of participants from other professions than dentistry. This might help to raise awareness of the high relevance of forensic odontology in DVI responses, which is currently under-appreciated [4]. Finally, encouraging general practice dentists to join the workshop might help to highlight the crucial importance of well-documented dental treatments, including imaging, to gain sufficient information for antemortem charting from clinical records.

## Conclusions

The DVI workshop described here used embalmed human remains and provided an opportunity to add dental identifications towards annual credentialling requirements for forensic odontologists. Participants rated the course to be excellent overall and highly relevant for their role. There is an interest to include further aspects of the DVI response such as fingerprinting or police work into the workshop as well as the inclusion of fragmentary remains, simulating the casework resulting from natural or physical causes that forensic odontologists can expect to encounter in the mortuary.

## Supplementary Information

Below is the link to the electronic supplementary material.

Supplementary file1 (PDF 243 KB)

Supplementary file2 (PDF 2579 KB)

Supplementary file3 (PDF 1782 KB)

Supplementary file4 (XLSX 16 KB)
