# Supplementary material for: A disaster victim identification workshop focused on forensic odontology using embalmed human remains
Source: Int J Legal Med. 2022 Mar 2;136(6):1801–9. doi: 10.1007/s00414-022-02790-5 (PMC9576667; doi:10.1007/s00414-022-02790-5)
Supplement: Supplementary file 1 — Supplementary file1 (PDF 243 KB) [file 414_2022_2790_MOESM1_ESM.pdf]

FORENSIC ODONTOLOGY CHARTING FOR DVI OPERATIONS

New Zealand Society of Forensic Odontology

1. To avoid confusion and to ensure consistency all dental annotations are to be in the FDI format and utilize the detailed charting conventions. Interpol Post Mortem Victim Identification Forms (PM) and Ante Mortem Victim Identification Forms (AM) are to be used. Typically the 100's, 600's and (as necessary) 700's pages of the AM and PM forms are completed. Annotations for every tooth in Section 630 (both AM and PM forms) are to be made (each line must have an entry). Annotations are also to be made on odontogram symbols as indicated.

2. All entries must be unambiguous. Use sections 635 (Specific Data) and 640 (Other Findings) and Page 700's to provide further information and description as necessary.

3. Charting should commence in the upper right jaw starting with tooth 18 and end with tooth 48. The tooth (FDI number), location / extent and material type is to be recorded as follows:

Surfaces (letters in capitals)

M = mesial
D = distal
V = vestibular (buccal, labial, facial)
L = lingual (palatal)
O = occlusal

Material (colour is used on the odontogram)

Amalgam = **AMF** — **BLACK**

Tooth coloured filling = **TCF** — **GREEN** — (composite, glass ionomer, porcelain)

Gold — **RED** — (semi-precious and precious)

4. The status of the teeth are to be recorded as follows;

a. **Sound tooth** (Tooth is intact – no caries or restorations. The terms 'intact' (INT) and NAD should not be used)

Descriptor	Abbreviate	Odontogram convention
Sound	**SOU**	No entry made on the odontogram

b. **Present tooth** (Tooth is present but no details are known about the condition of the tooth)

Descriptor	Abbreviate	Odontogram convention
Present	**PRE**	No entry made on the odontogram

c. **Missing tooth**

Descriptor	Abbreviate	Odontogram convention
Missing ante-mortem	**MAM**	• Place a **X** through the appropriate tooth symbol<br>**Note:** An empty socket where a tooth has been lost post-mortem is to be recorded as MPM
Missing post-mortem	**MPM**	• Place a circle ◯ around the appropriate tooth symbol

d. **Crown fracture** (The crown of the tooth is fractured with residual coronal material remaining)

Descriptor	Abbreviate	Odontogram convention
Crown fracture	**CRF**	• Outline extent of fracture on tooth symbol

e. **Residual root** (The crown of the tooth is lost (peri or post-mortem) leaving a residual root., includes teeth where the enamel crown has separated from the underlying dentine)

Descriptor	Abbreviate	Odontogram convention
Residual root	**RRX**	• Place a **#** symbol over the appropriate tooth symbol<br>**Note:** See comments regarding root fracture

f. **Root fracture** (A root or root fragment that has been left *in situ* ante-mortem – e.g. an incomplete extraction)

Descriptor	Abbreviate	Odontogram convention
Root fracture	**FRX**	• Place a black **FRX** over the appropriate tooth symbol

g. **Unknown** (No information available. It cannot be determined if a tooth/jaw/jaw portion was present or not)

Descriptor	Abbreviate	Odontogram convention
No information	**NON**	No entry made on the odontogram

h. **Space closed** (Tooth missing ante-mortem and all of the space for the tooth has been lost)

Descriptor	Abbreviate	Odontogram convention
Missing without diastema		• Use arrows over the appropriate tooth symbol →←

i. **Diastema** (Space opened)

Descriptor	Abbreviate	Odontogram convention
Diastema	**DIA**	• Use arrows over the appropriate tooth symbol ← →

j. **Tooth rotated**

Descriptor	Abbreviate	Odontogram convention
Tooth rotated	**ROT**	• Circle with arrow indicating direction of rotation over symbol ↻

k. **Un-erupted tooth**

Descriptor	Abbreviate	Odontogram convention
Unerupted tooth	**UNE**	• Place **UNE** over the appropriate tooth diagram

l. **Partially erupted tooth**

Descriptor	Abbreviate	Odontogram convention
Erupting	**ERU**	• Place **ERU** over the appropriate tooth diagram

m. **Impacted tooth**

Descriptor	Abbreviate	Odontogram convention
Impacted	**IMX**	• Place **IMX** over the appropriate tooth diagram<br>**Note:** Describe the nature of the impaction, eg. Mesio-angular

n. **Root filled tooth**

Descriptor	Abbreviate	Odontogram convention
Root filled	**RFX**	• Block out a triangle (in blue or black) on the lingual aspect of the appropriate tooth symbol. ▼

o. Other charting codes

Descriptor	Abbreviate	Odontogram convention
Caries	**CAR**	• Outline cavity in blue on the appropriate tooth symbol
Unfilled cavity	**CAV**	• Outline cavity in blue on the appropriate tooth symbol
Metal ceramic crown<br>(PFM, VMK, PFG)<br>Porcelain (ceramic) crown<br>Gold crown (FGC)	**MCC**<br>**POC**<br>**GOX**	• Colour tooth diagram with appropriate colour on the appropriate tooth symbol<br>**Note:** Information regarding crowns is to be recorded in the 'Specific Data' section 635 and 700
Bridge abutment<br>Bridge pontic	**ABU**<br>**PON**	• Abutments colour tooth diagram with appropriate colour, on the appropriate tooth symbol<br>• Pontic use a thick line from one abutment to the next over appropriate tooth symbol<br>**Note:** Information regarding bridges is to be recorded in the 'Specific Data' section 635 and 700
Implant	**IPX**	• Place IPX above the appropriate tooth symbol with the coronal restoration noted<br>**Note:** Information regarding implants is to be recorded in the 'Specific Data' section 635 and 700
Full upper denture	**FUD**	• Use 'brackets' and symbol FUD under the tooth symbols<br>**Note:** Information regarding dentures is to be recorded in the 'Specific Data' section 635 and 700
Full lower denture	**FLD**	• Use 'brackets' and symbol FLD above the tooth symbols.<br>**Note:** Information regarding dentures is to be recorded in the 'Specific Data' section 635 and 700
Partial upper denture	**PUD**	• Use the symbol PUD under the teeth included in the denture<br>**Note:** Information regarding dentures (material, clasp description etc) is to be recorded in the 'Specific Data' section 635 and 700
Partial lower denture	**PLD**	• Use the symbol PLD over the teeth included in the denture<br>**Note:** Information regarding dentures (material, clasp description etc) is to be recorded in the 'Specific Data' section 635 and 700

5. When charting DO NOT leave any blank spaces in the text boxes or on the odontogram.

Miscellaneous

6. When describing the dental material on the Post Mortem 600's form, single teeth are those that are clearly identifiable as teeth. Root and crown fragments should be recorded separately. Where the enamel cap of a tooth has been displaced from a root this should be recorded. These enamel caps are referred to as crowns; where an artificial crown is present it is referred to in accordance with the materials of construction (eg. MCC, POC). Root fragments and other tooth fragments are referred to as fragments.

7. Teeth that have been displaced from sockets are to be recorded as single teeth and not assigned to a tooth in the odontogram. If a tooth can be replaced in a socket and its fit can be verified radiographically then a note is to be made that the tooth can be repositioned in a specific tooth site.

If the charting information you require is not included above or if you do not know how to clearly record an observation seek guidance from the coordinator who is managing the DVI phase you are working in.

These conventions are to be used for DVI Operations		
Policy Facilitator: Hugh Trengrove	Version no.: 5	Issue date:21 Mar 2016

Acknowledgement: These guidelines are based on the Australian Society of Forensic Odontology SOP's developed in response the Victorian Bush Fires.
