# Supplementary material for: A disaster victim identification workshop focused on forensic odontology using embalmed human remains
Source: Int J Legal Med. 2022 Mar 2;136(6):1801–9. doi: 10.1007/s00414-022-02790-5 (PMC9576667; doi:10.1007/s00414-022-02790-5)
Supplement: Supplementary file 2 — Supplementary file2 (PDF 2579 KB) [file 414_2022_2790_MOESM2_ESM.pdf]

EXERCISE ONLY

Otago Daily Times

Friday, **29 October 2021**

Eight bodies recovered after Kawarau River plunge

39 0

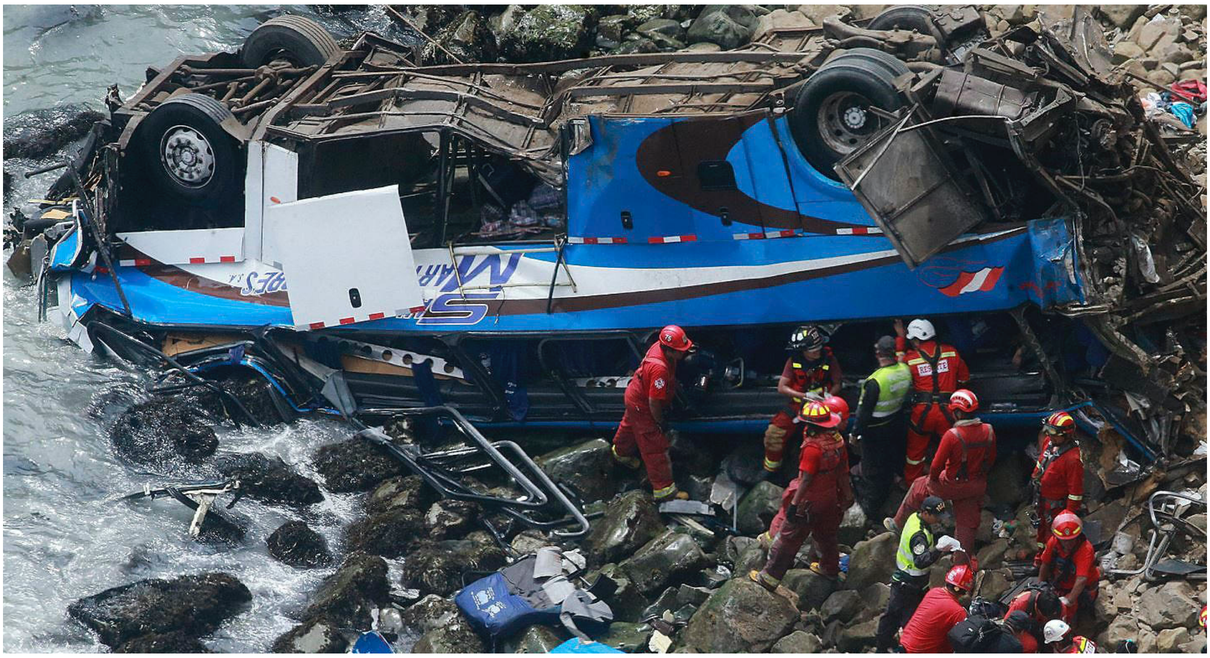

Rescue personnel work to recover bodies from the bus. Photo: Harriet Collins

Eight bodies have been recovered after a tour bus crashed into the Kawarau River near Queenstown last night. It is understood that there were 15 elderly passengers aboard from around Otago and Southland on a weekend cruise to Te Anau. The driver was a local man but has yet to be named.

"The Police Disaster Victim Identification team along with Search and Rescue personnel recovered the bodies from the partially submerged vehicle this afternoon," Otago Lakes central area commander Harry Jansen said.

"The victims are yet to formally identified and we have a number of further inquiries to carry out. A number of victims may have ended up in the gorge and have yet to be located".

EXERCISE ONLY
