# Supplementary material for: A disaster victim identification workshop focused on forensic odontology using embalmed human remains
Source: Int J Legal Med. 2022 Mar 2;136(6):1801–9. doi: 10.1007/s00414-022-02790-5 (PMC9576667; doi:10.1007/s00414-022-02790-5)
Supplement: Supplementary file 3 — Supplementary file3 (PDF 1782 KB) [file 414_2022_2790_MOESM3_ESM.pdf]

Tay Street Dental Centre
40 Tay Street Invercargill
Office Phone: 03 211 4890

Patient chart for Mrs Amber Harriet Rudd
Chart # 27/11/2020
SSN:
Birthdate: 13/04/1932 (89 years)

Entries thru 29-10-2021
Printed 29-10-21 at 9:46a

Patient Right

Patient Left

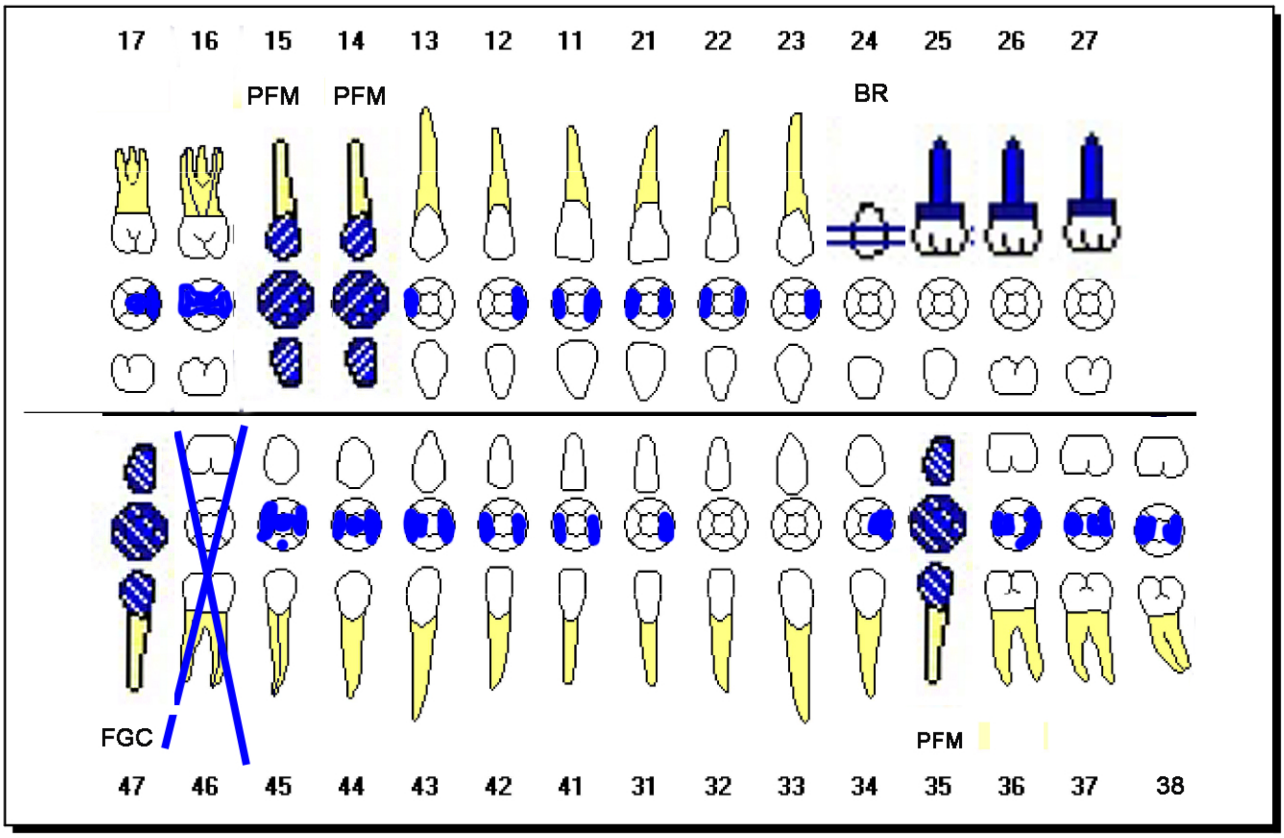

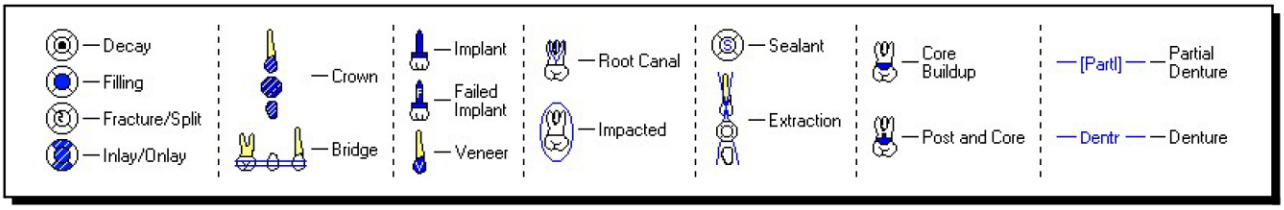

18-01-21	CG	Tooth #41	Concerned about chip in incisal edge, reassured.<br>Display character(s): cn
18-01-21	CG	Tooth #47	Tooth coloured filling on MOD (from ledger)
18-01-21	CG	Tooth #47	Results: Restoratives Used<br>EXCEPTIONS: Tetric Evo Ceram Bulk Fill Composite, Composite shades: IVB, Active prime and bond, Enamel Etch, Hypochlorite Clean
18-01-21	CG	Tooth #47	Results: LA Used<br>EXCEPTIONS: Local Anaesthetic Used: Scandonest 3% by IDB, Number of Cartridges: 1
18-01-21	CG	Entire mouth	Results: Covid Questions<br>EXCEPTIONS: Covid Questions Asked?<br>PBWs, PA 14, 35
18-01-21	SC	Unattached	NOTE: (cn) Hyg Notes<br>Tray 6150 24/11/20 110478<br>RFV: 3 mths<br><br>MH: Mild dementia. Meds: Atorvastatin, Aspirin, Methotrexate, Omeprasole, Celebrex, Donepezil Hydrochloride<br>Home cares: Green IDB<br>Perio condition: Looking after implants well<br>Tx: FM scl- hand and US FM prophy pol<br>OHI - advised patient to buy a soft TB when in the market for one next time. and to floss<br><br>Display character(s): cn
18-01-21	SC	Entire mouth	Removal of calculus - first visit (from ledger)
